# A sex-dependent role of Kv1.3 channels from macrophages in metabolic syndrome

**DOI:** 10.3389/fphys.2024.1487775

**Published:** 2024-11-13

**Authors:** Diego A. Peraza, Lucía Benito-Salamanca, Sara Moreno-Estar, Esperanza Alonso, José R. López-López, M. Teresa Pérez-Garcia, Pilar Cidad

**Affiliations:** ^1^ Departamento de Bioquímica y Biología Molecular y Fisiología, Universidad de Valladolid, Valladolid, Spain; ^2^ Unidad de Excelencia, Instituto de Biología y Genética Molecular (IBGM), Universidad de Valladolid y Consejo Superior de Investigaciones Científicas (CSIC), Valladolid, Spain

**Keywords:** bone marrow-derived macrophages, Kv1.3 channels, metabolic syndrome, cell migration, electrophysiology, macrophage phenotype, sex-dependent differences

## Abstract

**Introduction:**

Coronary artery disease (CAD) is the foremost single cause of mortality and disability globally. Patients with type 2 diabetes (T2DM) have a higher incidence of CAD, and poorer prognosis. The low-grade inflammation associated to T2DM contributes to increased morbidity and worst outcomes after revascularization. Inflammatory signaling in the vasculature supports endothelial dysfunction, leukocyte infiltration, and macrophage activation to a metabolic disease (MMe) specific phenotype, which could contribute to the metabolic disorders and ascular damage in T2DM. We have previously found that K_v_1.3 blockers inhibit the development of intimal hyperplasia, thereby preventing restenosis. This inhibition was enhanced in a mouse model of T2DM, where systemic K_v_1.3 blockers administration also improve metabolic dysfunction by acting on unidentified cellular targets other than vascular smooth muscle. Here we characterize the MMe phenotype in our T2DM model with a focus on macrophage K_v_1.3 channels, to explore their contribution to vascular disease and their potential role as targets to ameliorate T2DM vascular risk.

**Methods and Results:**

Male and female BPH mice fed on high-fat diet (HFD) develop metabolic syndrome (MetS) and T2DM. mRNA levels of several K^+^ channels (K_V_1.3, K_Ca_3.1, K_ir_2.1) and macrophage markers (TNFα, NOS2, CD36) were analyzed. The MMe phenotype associated with increased CD36 expression. Channel-specific fingerprinting highlights a gender-specific increase of K_V_1.3 mRNA fold change in LPS stimulated macrophages from HFD compared to standard diet (SD). K_V_1.3 functional expression was also significantly increased after LPS stimulation in female HFD macrophages compared to SD. Functional studies showed that macrophage's K_V_1.3 channels of BPH female mice did not contribute to phagocytosis or metabolic profile but were relevant in cell migration rate.

**Conclusion:**

Altogether, our data suggest that by inhibiting macrophage infiltration, Kv1.3 blockers could contribute to disrupt the vicious cycle of inflammation and insulin resistance, offering a novel approach to prevent MetS, T2DM and its associated cardiovascular complications in females.

## 1 Introduction

Type 2 diabetes mellitus (T2DM) significantly increases the risk of cardiovascular diseases (CVD), including coronary artery disease and restenosis following angioplasty or stenting. A key factor in the development of atherosclerotic and restenotic lesions is the abnormal proliferation and migration of vascular smooth muscle cells (VSMCs). Consequently, pharmaceutical interventions targeting VSMCs growth and migration represent a promising approach to treating T2DM-associated CVD.

Recent research has identified Kv1.3 channels in VSMCs as novel targets for treating undesirable vascular remodeling (Cidad et al., 2010; Cheong et al., 2011). Selective Kv1.3 blockade inhibits VSMCs remodeling and prevents restenosis in both animal models (Cidad et al., 2014; Bobi et al., 2020) and human vessels (Arévalo-Martínez et al., 2019). Notably, Kv1.3 inhibitors demonstrate increased efficacy in preventing restenosis in human T2DM vessels and in a mouse model of metabolic syndrome and T2DM (Arevalo-Martinez et al., 2021).

The benefits of Kv1.3 inhibition extend beyond vascular remodeling. In T2DM mice, Kv1.3 blockade reduces weight gain and associated inflammation, improves glucose tolerance, and eliminates insulin resistance (Upadhyay et al., 2013; Arevalo-Martinez et al., 2021). These beneficial effects are thought to result from increased energy expenditure and reduced obesity-induced inflammation in abdominal adipose tissue (Upadhyay et al., 2013). While Kv1.3 channels are known to play a crucial role in cytokine production and cholesterol accumulation in macrophages (Vicente et al., 2003; Yang et al., 2013), their specific contribution to macrophage function in T2DM remains unexplored.

Metabolic diseases like T2DM and obesity are characterized by low-grade chronic inflammation, leading to various complications (Hotamisligil, 2006). This inflammatory state is associated with the recruitment of pro-inflammatory monocytes and macrophages to various organs, including adipose tissue, liver, and blood vessel walls. The infiltration of macrophages into adipose tissue is a key initiating event in obesity-induced inflammation and insulin resistance. In conditions of over-nutrition, adipocytes secrete chemokines that attract monocytes to adipose tissue, where they differentiate into adipose tissue macrophages (ATMs). These pro-inflammatory ATMs, in turn, secrete additional chemokines, creating a self-perpetuating cycle of inflammation (Olefsky and Glass, 2010; Osborn and Olefsky, 2012). This feed-forward process significantly contributes to the progression of metabolic disorders and associated cardiovascular complications, although the functional involvement of macrophages in systemic metabolism is unclear (Hotamisligil, 2006).

Early studies attempting to classify macrophage inflammatory polarization as either inflammatory (M1) or anti-inflammatory (M2) oversimplified their complex and plastic biology. However, it is evident that macrophages in lean and obese tissues differ not only in number but also in functional properties. A plasma-membrane proteomic approach in both human and murine subjects, demonstrated that classical M1 inflammatory markers did not accurately define ATMs in obesity (Kratz et al., 2014), and a “metabolically-activated” macrophage (MMe) phenotype has been proposed to better characterize macrophages in high glucose/high fat environments. These pro-inflammatory ATMs exhibit a unique metabolic signature in obesity that promotes inflammatory cytokine release (Boutens et al., 2018). Similarly, fundamental differences in the regulation of genes linked to specific inflammatory triggers have been observed in bone marrow-derived macrophages (BMDM) (Robblee et al., 2016). Saturated fatty acid treatment of BMDM regulated the inflammasome, but the identity and expression time course of inflammatory mediators were distinct from those induced by the classical M1 pro-inflammatory stimulus, lipopolysaccharide (LPS), illustrating the complexity of macrophage activation in metabolic disorders. These observations highlight the need for a more detailed understanding of macrophage phenotypes in obesity and related metabolic conditions, beyond the simplistic M1/M2 dichotomy, which could potentially offer new targets for therapeutic interventions in obesity-related inflammation and its associated complications.

Here we characterized the phenotype of macrophages from a mouse model of MetS and T2DM (Arevalo-Martinez et al., 2021), using both BMDM (*ex vivo* differentiated from stem cells) and fresh peritoneal macrophages (*in vivo* differentiated). We explored the association between HFD-induced changes in the macrophages phenotype and changes in the functional expression of Kv1.3 channels, and we analyzed the potential impact of Kv1.3 changes in macrophage-integrated functions such as phagocytosis, migration and metabolism. Male and female mice were studied separately, as sex dependent differences in the low-grade chronic inflammation associated to metabolic diseases have been demonstrated before, and in some cases independently of steroid hormones (Gal-Oz et al., 2019; Li et al., 2023).

Functional expression of Kv1.3 channels was significantly upregulated in BMDM from HFD females (but not in males) upon LPS treatment. We did not find changes in oxygen consumption rate in BMDM from HFD females. In contrast, they displayed increased migration rate and phagocytic activity, but only migration rate was dependent on Kv1.3 expression and activity. As migration of pro-inflammatory macrophages into adipose tissue represents the initial step in obesity-induced inflammation and insulin resistance, our data suggest that inhibiting macrophage chemotaxis with Kv1.3 blockers could provide therapeutic benefits in MetS females without inhibiting other innate immune functions.

## 2 Material and methods

### 2.1 Animal model

Colonies of BPH/2J (blood pressure high) and BPN/3J mice (blood pressure normal, both from Jackson Laboratories, Bar Harbor, ME, United States) were housed in the animal facility of the School of Medicine of Valladolid, maintained by inbreeding crossing under temperature-controlled conditions (21°C) and with unlimited access to water and food. All procedures were approved by the Animal Care and Use Committee of the University of Valladolid (Project 505649), in accordance with the European Community guides (Directive 2010/63/UE).

To generate a mouse model of metabolic syndrome and type-2 diabetes mellitus (MetS/T2DM), 6 weeks old BPH mice were fed with a standard rodent chow diet (SD; Research Diets, #D12450J, 10% fat) or high-fat diet (HFD; Research Diets, #D12492, 60% fat) for 12 weeks. Weight was determined weekly, fasting blood glucose, cholesterol, insulin plasma level and blood pressure were obtained every 4 weeks and intraperitoneal glucose tolerance test (ipGTT), and intraperitoneal insulin tolerance test (ipITT) were determined every 6 weeks, as described elsewhere (Arevalo-Martinez et al., 2021).

### 2.2 Cell cultures

Animals were euthanized with isofluorane overdose (5%) in an anesthetic-gases chamber. BMDM were differentiated from mononuclear phagocytic precursor cells obtained after flushing bone marrow of femurs and tibiae of control (SD) or MetS/T2DM (HFD) BPH mice with RPMI. Precursor cells were propagated in suspension by culturing in RMPI supplemented with 10% FBS, 1% PSF, 1% L-glutamine and 10% L-929 supernatant in tissue-culture dishes. The precursor cells became adherent within 7–9 days of culture (Meana et al., 2014). Peritoneal macrophages (PM) were also obtained and cultured as previously described (Olivencia et al., 2021). PM were kept in culture overnight (16 h) in control media (control, resting macrophages) or in the presence of LPS (100 ng/mL; LPS-activated macrophages) before electrophysiological recordings.

### 2.3 RNA expression

Total RNA was extracted from BMDM culture with TRIzol® (Ambion). mRNA expression of K_V_1.3, K_Ca_3.1, K_ir_2.1, P2X_7_, TNF-α, NOS2 and CD36 (Table 1) was determined with qPCR with Taqman® and SYBR Green assays (Applied Biosystems) using RPL18 (ribosomal protein L18) as housekeeping (Arevalo-Martinez et al., 2019). qPCR was performed in a Rotor-Gene 3,000 instrument, and the relative quantification method (2^−ΔΔCT^) was used to express mRNA levels (Livak and Schmittgen, 2001; Cidad et al., 2010). The information regarding commercial assays and/or primers sequences is listed in Supplementary Table SI.

**TABLE 1:** mRNA expression levels of several phenotype markers (upper rows) and ion channels (lower rows) were determined with qPCR in BMDM from male and female mice subjected to SD or HFD. mRNA levels are expressed as normalized abundance (2^−ΔCt^) using Rpl18 as the housekeeping gene. Statistical comparisons were carried out using two-way ANOVA followed by Tukey’s test in the case of normal distributions and equal variances; alternatively Kruskal-Wallis analysis followed by Dunn’s test was used. Red numbers indicate p-values of significant differences compared to SD, and blue numbers indicated the p-value for the significant differences between males and females. Values are mean ± SEM of 8–12 triplicate determinations, obtained from two batches of SD and HFD mice in each group (male and female). A graphical representation of these data is available in the supplemental file.

Gen	Sex	SD-control	HFD-control	SD-LPS fold change	HFD-LPS fold change
Tnf-α	Female	1.28 ± 0.16	1.31 ± 0.11	6.31 ± 1,99	5.01 ± 1.89
	Male	0.42 ± 0.07	0.38 ± 0.04 (0.0001)	1.91 ± 029	2.71 ± 0.33
Nos2	Female	2.91E-03 ± 0.97E-03	1.56E-03 ± 0.37E-03	113 ± 30,82	153.9 ± 45.8
	Male	0.335E-03 ± 0.88E-03	0.34E-03 ± 0.076E-03	99.54 ± 17.03	107.92 ± 30.08
Cd36	Female	37.53 ± 4.97	53.48 ± 4.36 (0.011)	0,25 ± 0,03	0.24 ± 0.05
	Male	22.71 ± 3.07	18.96 ± 3.08 (0.000)	0.25 ± 0.061	0.28 ± 0.05

Gen		SD-control	HFD-control	SD-LPS fold change	HFD-LPS fold change
Kcna3	Female	2.19E-03 ± 0.22E-03	1.43E-03 ± 0.15E-03	8,04 ± 1,41	20,18 ± 4,48 (0.025)
	Male	1.88E-03 ± 0.17E-03	1.86E-03 ± 0.27E-03	2.23 ± 0.44	3.11 ± 0.48 (0.000)
Kcnn4	Female	1.52 ± 0.15	1.12 ± 0.13	0.62 ± 0.13	0.59 ± 0.07
	Male	1.15 ± 0.18	1.05 ± 0.07	0.36 ± 0.045	0.30 ± 0.038 (0.03)
Kcnj2	Female	25.06E-03 ± 1.9E-03	55.9E-0.3 ± 6.3E-03 (0.000)	0.4 ± 0.12	0.19 ± 0.02 (0.02)
	Male	30.35E-03 ± 0.19E-03	60.59E-03 ± 4.8E-03 (0.000)	0.13 ± 0.037 (0.003)	0.06 ± 0.011 (0.03)
P2rx7	Female	0.297 ± 0,033	0.299 ± 0,024	1.05 ± 0,11	1.07 ± 0,16
	Male	0.170 ± 0.012	0.194 ± 0.02	0.94 ± 0.20	0.92 ± 0.20

### 2.4 Electrophysiological methods

Ionic currents were recorded at room temperature using whole-cell configuration of the patch clamp technique as previously described (Lordén et al., 2017; Cidad et al., 2020; Olivencia et al., 2021). BMDM and PM were plated on the bottom of a small recording chamber (0.2 mL) on the stage of an inverted microscope and perfused by gravity. The composition of the bath solution was (in mM): 141 NaCl, 4.7 KCl, 1.2 MgCl_2_, 1.8 CaCl_2_, 10 glucose, and 10 HEPES, pH 7.4 with NaOH. Recording pipettes were pulled to obtain resistances ranging from 2 to 4 MΩ when filled with an internal solution containing (in mM): 125 KCl, 4 MgCl_2_, 10 HEPES, 10 EGTA, 5 MgATP, pH 7.2 with KOH.

Total current amplitude was explored with either 500 ms voltage ramps from −140 to +60 mV every 5 s or current/voltage (I/V) curves with 200 ms pulses from −80 to +60 mV in 10 mV steps. C-type inactivation was explored by analyzing use-dependent block, using trains of pulses of 250 ms duration from −80 to +40 mV at a frequency of 2 Hz. Pharmacological characterization of the currents was carried out by recording ramps after perfusing the cells with bath solutions containing the Kv1.3 blocker PAP-1 (100 nM, Sigma #P6124), the K_Ca_3.1blocker TRAM-34 (100 nM; Sigma # T-6700) the nonselective K channel blocker TEA (5 mM, Sigma, # 177806), or a solution containing 100 µM Ba_2_Cl to block inward rectifier K^+^ currents (Miguel-Velado et al., 2005; Cidad et al., 2010; Tajada et al., 2012). Purinergic currents were obtained in continuous recording from a holding potential of 0 mV, by perfusing macrophages with an external solution containing 2 mM ATP (Sigma # A-2383) or the specific P2X_7_ activator-BzATP (700 μM; Alomone #: A-385).

### 2.5 Phagocytic activity

Phagocytosis was assessed using zymosan A fluorescent particles and flow cytometry. Briefly, BMDM were detached with 5 mM EDTA in HBSS for 30 min at 37°C in a humidified 5% C0^2^ incubator and 2.5 × 10^5^ cells/condition were resuspended in PBS with the corresponding treatments. LPS (100 ng/mL) and PAP-1 (200 nM) were added 18 h and 30 min before the addition of zymosan respectively. Alexa Fluor 594 zymosan A (Molecular probes) particles were added to the cell suspension at a ratio of 1:5 (particles:cells). The mixture was incubated at 37°C for 15 min in a humidified 5% C0_2_ incubator to allow phagocytosis to occur. Following incubation, cells were washed twice with cold PBS to remove non-phagocytosed particles and fixed using 4% paraformaldehyde for 10 min. Cells were kept at 4°C before and after the 15 min incubations to avoid unwanted phagocytosis. Samples were then analyzed using an Aurora flow cytometer (Cytek Biosciences). The percentage of Alexa Flour-positive cells was determined using Kaluza Analysis 2.1 software (Beckman Coulter). Negative controls (cells without zymosan) were included in each experiment.

### 2.6 Seahorse Cell Mito Stress Test

The oxygen consumption rate (OCR) was measured in real-time using a Seahorse XF24 Analyzer (Agilent, California, CA, United States) with Seahorse XF Cell Mito Stress Test Kit (Agilent, cat#: 103015-100), following manufacturer’s instructions, using the respiratory chain inhibitors oligomycin (10 μM), FCCP (25 μM), rotenone (10 μM) and antimycin A, (25 μM). The seahorse analyzer was calibrated with a calibrating Seahorse XF solution (Agilent, 102342-100).

Briefly, BMDM were seeded in 24-well Seahorse assay plates at a concentration of 4 × 10^4^ cells/well and cultured overnight with control media or media with 100 ng/mL LPS 100 nM PAP-1 was also present in some experimental groups of control or LPS-treated cells overnight. Next day, macrophages were washed, and the medium was replaced with Seahorse XF RPMI (Agilent, cat#: 103576-100) supplemented with 10 mM glucose (Agilent, cat#: 103577-100), 1 mM pyruvate (Agilent, cat#: 103578-100) and 2 mM glutamine (Agilent, cat#: 103579-100). Hoechst (Invitrogen, H3570) was used to evaluate cell viability and normalize readings from the Seahorse XF Analyzer.

### 2.7 Migration assays

Scratch migration assay was carried out in confluent BMDM cultures plated in 24 well plates. BMDM were incubated for 24 h in serum-free media, and LPS-stimulated macrophages were incubated the last 16 h with 100 ng/mL LPS. Next day, after creating a wound in the macrophage monolayer with a 10 µL tip, the medium was refreshed and 100 nM PAP-1 was added to some wells. Images of the same areas were taken at different time points (from 0 to 12 h) and ImageJ (Fiji) software was used to calculate the fraction of free area (normalized area) as A_X_/A_0_, where A_0_ is the area at t = 0 and A_X_ is the area at the selected time point. The area under the curve (AUC) in each condition was calculated and used for statistical comparisons among conditions.

### 2.8 Statistical analysis

Statistical analysis was carried out using Origin 2023b and Microsoft Excel software. The combined data were presented as mean values ±standard error of the mean (SEM) derived from multiple experiments. P-values<0.05 were considered significantly different. For comparisons between two groups with normal distribution, Student’s t-test, for paired or unpaired data as required, was used to determined p-values. For comparisons among several groups, one-way, two-way or three-way ANOVA followed by Tukey’s test was employed in the case of normal distributions and equal variances; alternatively Kruskal-Wallis analysis followed by Dunn’s test was used. Shapiro-Wilk test and Levene´s or Bartlett’s test were used to test normality and homogeneity of variances respectively.

## 3 Results

### 3.1 Characterization of the MetS/T2DM mouse model in male and female

**FIGURE 1:**
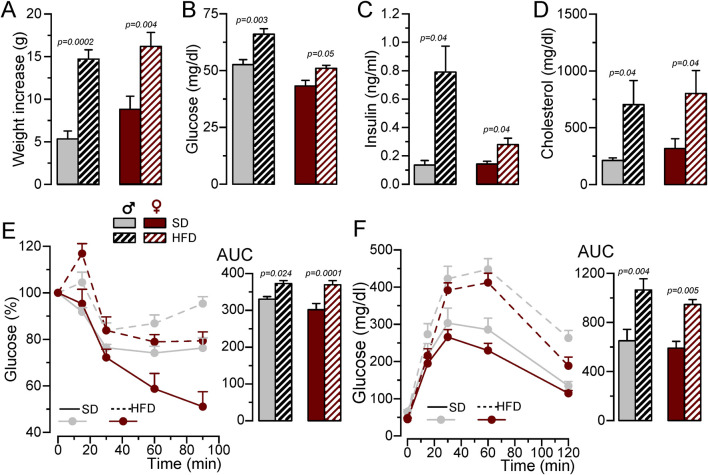
Generation of a MetS/T2DM mouse model. **(A)**. Average weight gain of BPH/SD and BPH/HFD male (grey) and female (red) mice. Mean ± SEM data from 8 to 12 mice per group, Kruskal-Wallis analysis followed by Dunn´s test and Student’s t-test, respectively, were used to estimate significance between SD and HFD. **(B)**. Fasting blood glucose levels after 12 weeks in SD or HFD; n = 8-12 animals in each group; Kruskal-Wallis analysis followed by Dunn´s test (for male data) and and Student’s t-test (for female). **(C)**. Basal insulin blood levels determined with an ELISA assay, n = 4-5 animals per group Kruskal-Wallis analysis followed by Dunn´s test. **(D)**. Blood cholesterol levels determined by spectrophotometry at the end of the 12-week treatment with SD or HFD. Student’s t-test to estimate mean differences, n = 5-11 per group. **(E)**. IpGTT after 12 weeks of SD or HFD, male BPH/SD (n = 9), male BPH/HFD (n = 11, female BPH/SD (n = 8) and female BPH/HFD (n = 12), both the time course of blood glucose level after glucose overload in fasting animals and the area under the curve (AUC) are represented, p-values are from Student’s t-test. **(F)**. IpITT after 12 weeks of HFD obtained from the same animals as in **(E)**. The plots show the time course of changes in blood glucose (100% = glucose at t = 0) after insulin load, and the area under the curve (AUC), p-values are from Student’s t-test.

To develop a MetS/T2DM model, 6 weeks old BPH male or female mice were fed with SD or HFD for 12 weeks. BPH mice are hypertensive as compared to their controls BPN (Mean BP values in male were BPN = 70.39 ± 3.8 mmHg and BPH = 101.19 ± 1.3 mmHg and in female BPN = 75.16 ± 1.9 mmHg and BPH = 95.38 ± 2.1 mmHg), and HFD did not induce significant changes in BP (mean BP in HFD male = 101.01 ± 2.9 mmHg and in HFD female = 97.67 ± 3.8 mmHg). However, mice fed with HFD exhibited significant weight gain (Figure 1A), and developed an increase in fasting glucose, basal insulin levels and plasma cholesterol (Figures 1B–D), together with glucose intolerance and insulin resistance (Figures 1E, F).

### 3.2 BMDM phenotype in MetS/T2DM model in male and female

**FIGURE 2:**
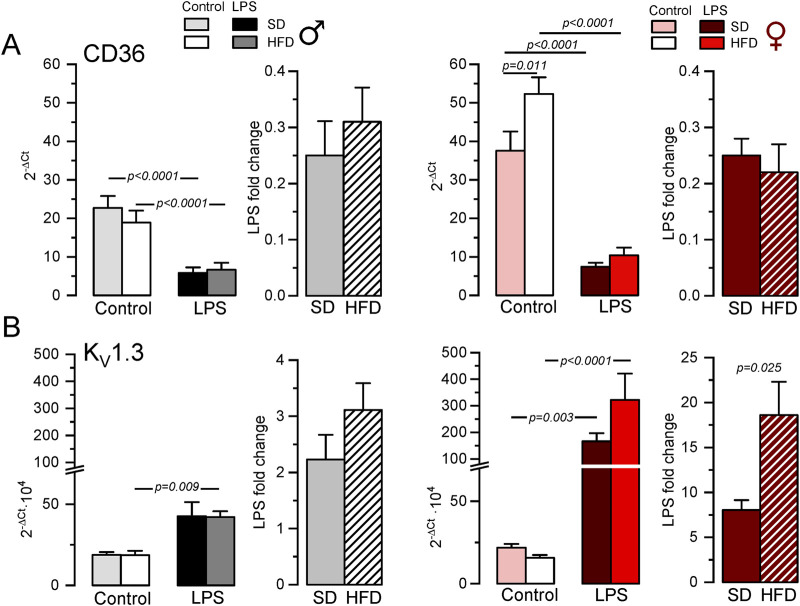
Gene expression changes in BMDM from the MetS/T2DM mice model. **(A)** mRNA expression levels of CD36 were determined in male (left plot, grey scale colors) and female (right plot, red scale colors) BMDM obtained from SD and HFD-fed mice. In both groups, mRNA expression levels under resting conditions (control) and after LPS treatment for 16 h (LPS) were obtained, and the differences between these two conditions are plotted as LPS-fold change. mRNA values were normalized using Rrl18 as housekeeping gene and expressed as 2^−ΔCt^. Data are mean ± SEM of 8–12 triplicate determinations, obtained from two batches of SD and HFD mice in each group (male and female). P-values were obtained with a two-way ANOVA followed by Tukey´s post-hoc test. **(B)**. The same analysis as in A was carried out for mRNA expression levels of KCNA3 gene (Kv1.3), with mRNA data obtained from the same samples. Kruskal-Wallis analysis followed by Dunn’s test was used to estimate significance between control and LPS-treated BMDMs mRNA levels, and one-way ANOVA followed by Tukey´s post-hoc test was used for LPS-fold change data.

Next, we explored the phenotypic changes in BMDM obtained from our HFD mice. mRNA expression profile of markers of pro-inflammatory macrophages (TNF-α, NOS2) or MMe metabolically-activated macrophages (CD36; Kratz et al., 2014) were determined in male and female BMDM both at rest and after LPS treatment (Supplementary Table SI; Supplementary Figure SI). While TNFα and NOS2 increased in pro-inflammatory macrophages, LPS treatment decreased CD36. No significant sex-dependent differences in these markers could be observed in control (SD) conditions, but upon HFD levels of TNFα and CD36 were significantly higher in female compared to male. However, the only diet-induced change in these markers was the upregulation of CD36 in control BMDM in HFD females (Figure 2A). The upregulation of CD36 in these conditions was confirmed at the protein level using flow cytometry (Supplementary Figure SII)

Many functional ion channels have been described in macrophages, and some of them can be regarded as potential therapeutic targets in several immune disease, as they have been shown to contribute to macrophage polarization towards pro-inflammatory or anti-inflammatory phenotype also referred as M1 or M2 respectively. The expression levels of mRNA encoding for some previously described K^+^ channels (which can also serve as phenotypic markers), were determined in BMDM. LPS treatment led to upregulation of Kv1.3 mRNA (Kcna3, Figure 2B) and concomitant downregulation of Kir2.1 mRNA [Kcnj2 (Vicente et al., 2003; Chen et al., 2022), Supplementary Table SI]. We also found a LPS-induced downregulation of K_Ca_3.1 (Kcnn4), whose role in macrophage polarization is more controversial (Xu et al., 2017). Finally, we explored the expression levels of the ATP-activated receptor P2X_7_ (P2rx7) that has been proposed as a marker of MMe phenotype (Kratz et al., 2014), although its functional contribution is not clearly established (Sun et al., 2012). With the exception of P2X_7_, which showed no sex- diet- or LPS-induced changes in mRNA expression, LPS-fold changes in HFD were always significantly larger in female compared to male BMDMs (Table 1, p-values in blue). Moreover, HFD treatment led to a significant increase in Kcnj2 expression in both male and female in control, unstimulated BMDMs, with no significant changes in Kcna3 and Kcnn4 expression. In the case of Kcna3 in females, HFD appears to both reduce its expression in control and enhance LPS-induced upregulation, leading to a significant diet-induced fold change increase upon LPS activation (Figure 2B).

### 3.3 Modulation of outward K^+^ currents in macrophages from MetS/T2DM model in male and female

**FIGURE 3:**
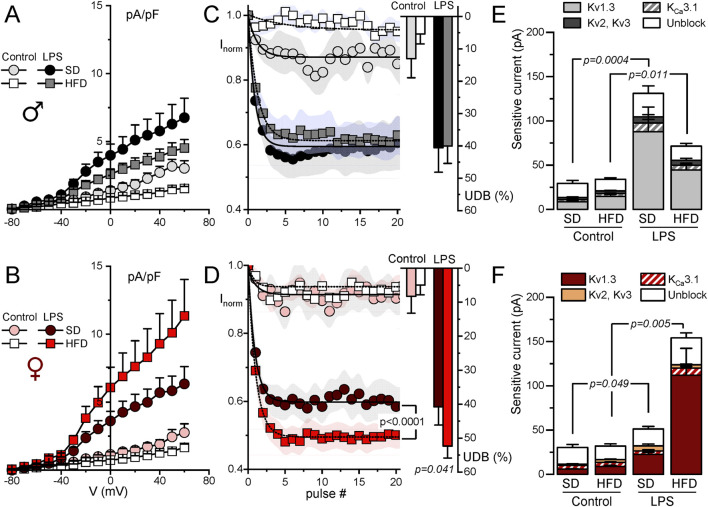
Electrophysiological characterization of outward K^+^ currents from BMDM. Kinetic and pharmacological studies were carried out in BMDM obtained from female (red scale colors, lower plots) and male mice (upper plots, grey scale colors). In all cases, BMDM obtained from SD and HFD-fed mice were studied at rest (control) and after16 h treatment with 100 ng/mL LPS (LPS) as indicated. **(A, B)**. Current density to voltage relationships were constructed from peak outward current amplitudes obtained in 200 ms pulses from −80 to +60 mV in 10 mV steps, from a holding potential of −80 mV. Data are mean ± SEM of 15–25 cells in each group, obtained from at least six different animals. **(C, D)**. Peak current amplitude was obtained from trains of 40 pulses of 250 ms from −80 mV to +40 mV applied every 0.5 s, and normalized to the amplitude of the first pulse. The symbol graphs show the mean ± SEM of the first 20 pulses fitted to a one-exponential decay function, and the bars plots on the right were obtained by averaging the normalized amplitude (expressed as %) of the last 20 pulses in each group. P values were obtained from F-test comparison between the fits (for the normalized current plot) and with one-way ANOVA for the % of UDB. Data are mean ± SEM of 10–20 cells per group, from at least 5 different cultures. **(E, F)**. Average peak current amplitude for the PAP-1 sensitive (Kv1.3 current), the TRAM34-sensitive (K_Ca_3.1 current) the 5 mM TEA-sensitive (Kv2-Kv3 current) and the insensitive outward K current (unblock) in each of the conditions. 7–11 BMDM obtained from at least 4 different animals were used for each determination Statistical analysis was carried out with Kruskal-Wallis analysis followed by Dunn’s test to compare the fraction of Kv1.3-sensitive current in each condition.

The functional contribution of the changes in K^+^ currents was next explored with electrophysiological techniques both in BMDM and in fresh PM from SD and HFD mice. The summary data obtained from male and female BMDM are shown in Figure 3. In control macrophages, K^+^ current amplitudes were smaller in HFD BMDM in both sexes (Figures 3A, B). However, in LPS-treated macrophages the effect of HFD was sex dependent, and current density was smaller in males and larger in females. These changes in female BMDM are in good agreement with the observed differences in Kv1.3 mRNA expression levels (Figure 2B). To further confirm this extent, kinetic and pharmacological analysis of these currents was carried out. Kv1.3 channels exhibit a characteristic C-type inactivation (Vennekamp et al., 2004; Kurata and Fedida, 2006) that translates into a use-dependent block (UDB) upon repetitive stimulation. This UDB was taken as a kinetic parameter to explore Kv1.3 contribution to total outward K^+^ currents in macrophages (Figures 3C, D). No significant differences were found in control macrophages, but LPS-stimulated BMDM from HFD females (but not males) showed a significant increase of UDB compared to SD BMDM, suggesting an increased Kv1.3 functional expression in these cells. Finally, a pharmacological dissection of the outward K^+^ currents was performed in a group of cells, using sequential application of PAP-1 (100 nM) to block Kv1.3 currents, TRAM34 (100 nM) to block K_Ca_3.1 and TEA (5 mM) to block Kv2 and Kv3 currents. Kv1.3-sensitive current represented the largest fraction of K^+^ current in all cases, and the main contributor to the outward current upregulation of LPS-treated BMDM (Figures 3E, F). The increase of the Kv1.3-current fraction in LPS-treated macrophages parallels the data shown in A and B, being significantly larger in HFD BMDM from females compared to SD. In contrast, a reduction of Kv1.3 currents in HFD macrophages stimulated with LPS was observed in male BMDM.

**FIGURE 4:**
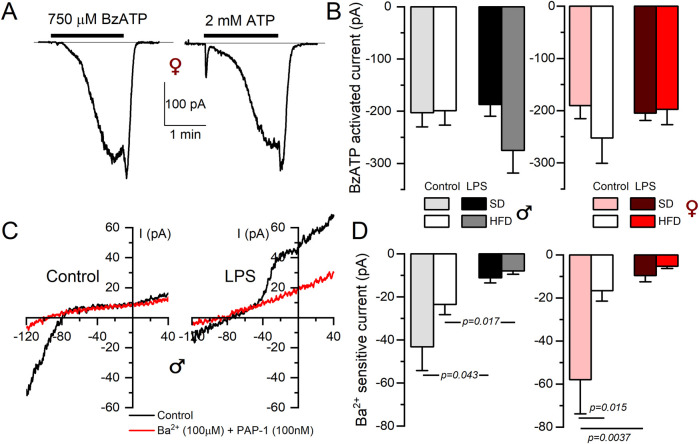
Electrophysiological characterization of P2X_7_ and inward rectifier K^+^ currents from BMDM. **(A)** Representative currents obtained from a female control BMDM upon application of either BzATP (750 µM) or 2 mM ATP during the indicated time to show the kinetics and amplitude of the P2X7-mediated currents. Holding potential was kept at 0 mV. **(B)** The right panels show de average current amplitude elicited by 750 µM BzATP in BMDM from the indicated groups obtained from female (red scale) or male (gray scale) mice. Each column is mean ± SEM of 12–20 cells in each group, obtained from 4 to 6 different animals. A two-way ANOVA did not show significant differences. **(C)** Representative recordings of the current elicited in one control and one LPS-treated BMDM SD male with voltage ramps from a holding potential of −80 mV with control solution (black traces) or in the presence of a baths solution containing 100 nM PAP-1 and 100 µM BaCl_2_ to block Kv1.3 and Kir2.1 currents respectively (red traces). **(D)** The amplitude of the BaCl_2_-sensitive current was measured at −120 mV, and the averaged values obtained in all experimental conditions are plotted in the right graphs, both in female (red color scale) and male (gray colors) BMDM. Statistical analyses were carried out with a two-way ANOVA followed by Tukey´s post-hoc test (in the females group) and with a Kruskal-Wallis analysis followed by Dunn’s test in the males group. Mean ± SEM, n = 8-12 cells per group from at least 4 different cultures.

Electrophysiological characterization of BMDM also confirm expression data for P2X_7_ receptors (Figures 4A, B). BzATP is a P2X_7_ receptor agonist used to explore the activity of these currents. As shown in the figure, we did not find any change BzATP-activated currents dependent either on macrophage activation or sex. However, discrepancies between mRNA expression and protein function (determined by electrophysiological recordings) were found in the case of inward rectifier K^+^ currents (Kir2.1 channels). Figure 4C shows the effect of 100 µM BaCl_2_ and PAP-1 on K^+^ currents obtained in male BMDM in SD from resting (control) and LPS-stimulated macrophages. A decrease of inward currents in LPS-stimulated BMDM is evident. However, with regards to diet, while mRNA shows a significant upregulation upon HFD treatment, (see Table 1), we found a tendency to a decreased BaCl_2_-sensitive current when comparing control SD versus control HFD in both sexes (Figure 4D), that reached statistical significance in female BMDM.

The characterization of the outward K^+^ currents was also carried out in peritoneal macrophages (PM) (Supplementary Figure SIII). As in BMDM, outward K^+^ currents in control PM were decreased in HFD-fed mice (figure IIA and D), most likely due to a decreased contribution of Kv1.3, as suggested by the significant reduction of UDB in these conditions, both in male and female (Figure IIB y E). The activation with LPS elicited larger increases in Kv current density in female than in male PM, but in both cases were reduced in HFD-treated PM. Altogether, the electrophysiological characterization of PM did not show the Kv1.3 changes observed in female BMDM from HFD. However, in this latter preparation changes expression and function of Kv1.3 channels exhibit a good correlation.

### 3.4 MetS/T2DM female BMDM show Kv1.3-independent increased phagocytosis

**FIGURE 5:**
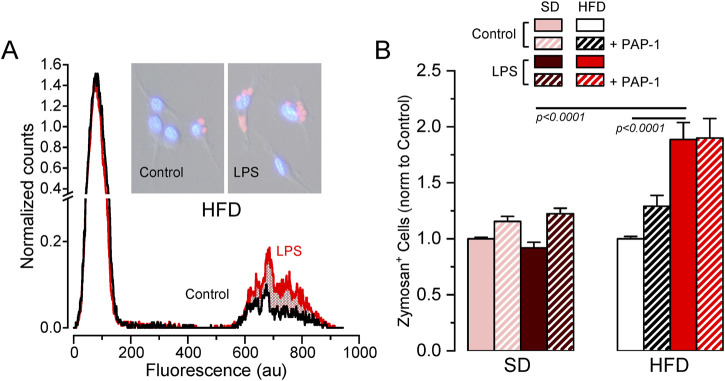
Effects of HFD on female BMDM phagocytosis. **(A)**. Example of a flow cytometer experiment in a control (black line) and LPS-treated (red line) BMDM population from a HFD female to determine phagocytosis. The graph shows the normalized counts for each cell population (obtained by normalizing the running area integral of each experiment, to correct for the total number of cells), against fluorescence intensity. An increase in the number of cells showing fluorescence from Alexa Fluor 594-labelled zymosan was observed in HFD LPS-treated cells (red area). Representative pictures of control and LPS cells with labelled zymosan particles are also shown. **(B)**. Average data (mean ± SEM) obtained from 4-7 independent experiments in each condition, carried out with BMDM from different mice. In this case, the percentage of zymosan-labelled cells was normalized to its own control, untreated cells in each experiment. Statistical significance was obtained with a three-way ANOVA followed by Tukey´s post-hoc test.

As the previous data exploring HFD-associated changes in Kv1.3 channels and metabolic markers indicated sex-dependent significant differences only in female mice, we focused on female BMDM to further explore the functional impact of these changes in several macrophage´s integrated responses. We studied phagocytosis using flow cytometry to measure the number of cells that uptake Alexa Fluor 594-labelled zymosan A particles (Figure 5). In SD BMDM, there was no difference in the number of zymosan-labelled cells upon LPS treatment. In contrast, LPS-stimulated HFD cells showed increased phagocytosis compared to LPS-stimulated SD or control HFD macrophages. However, Kv1.3 blockade had no effect in any condition, indicating that the increased phagocytic capacity of LPS-activated BMDM from MetS/T2DM female mice is independent of Kv1.3 channels.

### 3.5 BMDM from MetS/T2DM females did not show changes in O_2_ consumption rate

**FIGURE 6:**
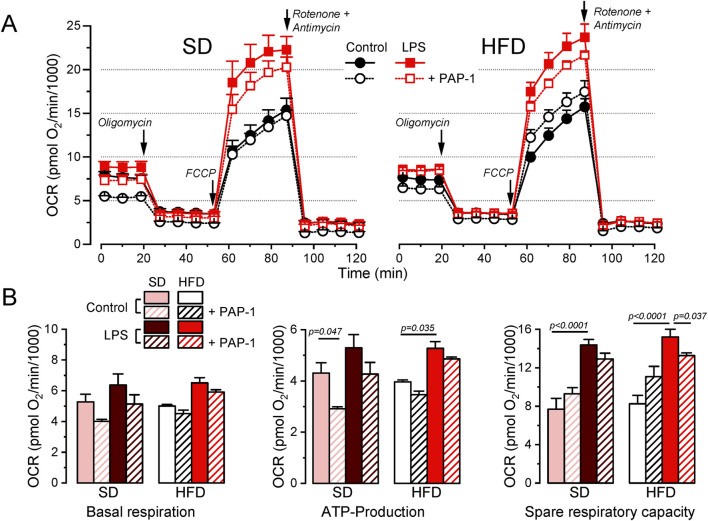
Metabolic profile of SD and HFD BMDM from female mice. **(A)**. Average data from Seahorse XF Cell Mito Stress Test experiments carried out in BMDM from SD (left) and HFD (right) female mice, using resting (control) and activated (LPS) cells with or without overnight treatment with 100 nM PAP-1 as indicated. The Cell Mito Stress Test profile was obtained by sequential application of the drugs indicated (see methods for details). Data was normalized by counting of cells and expressed as pmol O_2_/min/1,000 cells. **(B)**. Quantification of several standard parameters in the Seahorse analysis. The plots show the average (mean ± SEM) data of OCR in basal respiration (before oligomycin application), ATP-linked respiration (the difference in OCR between basal respiration and oligomycin) and spare respiratory capacity (OCR difference between basal and FCCP). Data were obtained from 3 independent experiments, each one containing 3 replicates and analyzed with a 3-way ANOVA followed by Tukey´s post-hoc test.

Potential changes in energy metabolism between BMDM from SD and HFD female mice were explored using a Seahorse analyzer to study oxygen consumption rate (OCR). We assessed OCR in control media and after the sequential addition of oligomycin (ATP synthase inhibitor), carbonyl cyanide 4-(trifluoromethoxy)phenylhydrazone (FCCP, a H^+^ ionophore) and rotenone/antymicin A (complex I and III inhibitors respectively) to calculate basal respiration, ATP-linked respiration and spare respiratory capacity. Average data obtained in both groups, analyzing also the effects of LPS activation and PAP-1 treatment are depicted in Figure 6. We found no differences in basal respiration among the different experimental conditions. The analysis of the ATP-linked respiration indicates that this parameter is dependent on Kv1.3 in resting (control) macrophages in SD but this dependence is lost in HFD, which is consistent with the reduced functional expression of Kv1.3 channels after HFD treatment (Figure 3). However, ATP-linked respiration increases significantly upon LPS-activation in HFD, matching Kv1.3 upregulation, but in this case, it is PAP-1 insensitive, suggesting a change in the mechanism(s) involved in respiration in activated BMDMs. The spare respiratory capacity was increased after LPS stimulation in both BMDM from SD and HFD and it was Kv1.3-dependent only in HFD macrophages.

### 3.6 BMDM from MetS/T2DM females show Kv1.3-dependent increased migration

**FIGURE 7:**
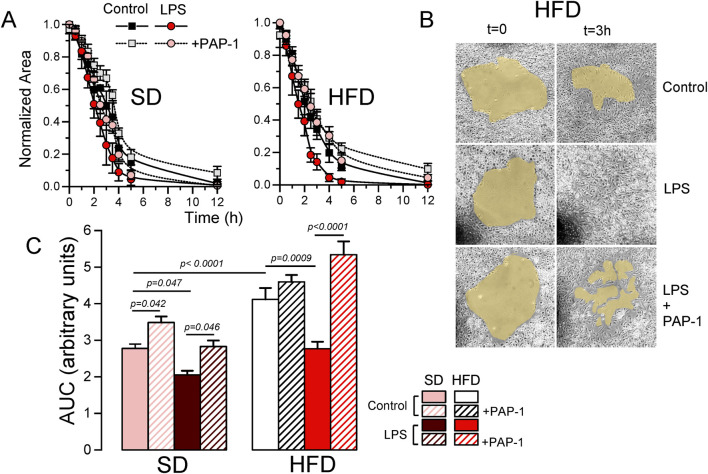
Effects of HFD on the migration of BMDM from female mice. **(A)**. Representative examples of a migration experiment using BMDM from SD (left) or HFD (right) female mice. In each experiment the four indicated conditions (Control and LPS-treated with or without PAP-1) were analyzed using three independent replicates. The time course of the invaded area up to 12 h was determined at the indicated times, to obtain the area free of cells, which was normalized to the initial area. **(B)**. Sample images obtained at t = 0 and after 3 h in control, LPS and LPS + PAP-1 in one experiment carried out with BMDM from a HFD female mouse. **(C)**. The bars plot represents the averaged area under the curve obtained in 4-6 independent experiments as the ones shown in A, each one from a different cell culture. Statistical significance was obtained from a three-way ANOVA followed by Tukey´s post-hoc test.

A key to an efficient and optimally regulated macrophage´s response is their ability to shift between a resting and activated mobile state rapidly, combined with stringent regulation of cell migration during the activated state. Consequently, mechanisms controlling macrophage mobility and migration play a key role in the efficiency of their immune or inflammatory responses. For this reason, we explore the role of Kv1.3 channels in the migration of BMDM from both SD and HFD female mice (Figure 7). As in the previous experiments, PAP-1 treatment was used to infer Kv1.3 contribution. The representative plots (Figure 7A) show the time course of the changes in the invaded area in experiments in SD and HFD BMDM in all the conditions tested. Control BMDM migration was inhibited by PAP-1 in SD cells but not in HFD cells. LPS-stimulated BMDM exhibit an increased migration rate (compared to control) that was sensitive to PAP-1 in both groups. The averaged area under the curve (AUC) obtained in pooled experiments confirmed these results (note that larger AUCs represent slower migration rates). These data suggest that differences in migration rate between SD and HFD macrophages are dependent on Kv1.3 expression levels, as the magnitude of PAP-1 effects shows a good correlation with Kv1.3 expression levels in each condition (Figure 7B).

## 4 Discussion

### 4.1 Macrophage phenotype contribution to metabolic syndrome and T2DM

The metabolic syndrome (MetS) is a cluster of clinical disorders including central obesity, dyslipidemia, glucose intolerance and hypertension. It is known that these disorders not only increase the chances of developing T2DM, but also Cardiovascular diseases (CVD). Insulin resistance has been considered at the root of the problem to explain the metabolic abnormalities within this syndrome, but new evidence points to several pro-inflammatory cytokines, reactive oxygen species and free fatty acid intermediates as key elements in sustaining and perpetuating the development of the MetS and its CVD complications.

Macrophages are a well-established key player in cardiovascular disease, particularly in atherosclerotic plaque formation and remodeling (Aparicio-Vergara et al., 2012; Simon-Chica et al., 2022). Recent findings show that they also accumulate in adipose tissue of obese mice, contributing to chronic low-grade inflammation and disrupting glucose and lipid metabolism leading to MetS and CVD. The mechanism involves mononuclear cells migrating to white adipose tissue, releasing pro-inflammatory cytokines, and promoting insulin resistance in skeletal muscle, liver, and other tissues (Donath and Shoelson, 2011). Clinical observations reveal that MetS subjects show higher circulating levels of inflammatory cytokines and greater macrophage infiltration compared to healthy controls (Andersen, et al., 2016). Notably, deletion of the insulin receptor in myeloid cells reduces macrophage infiltration during HFD, decreases circulating levels of TNF-α and protects against HFD-induced insulin resistance, highlighting insulin’s potential negative role in innate immune responses during MetS.

### 4.2 Kv1.3 channels contribution to metabolic syndrome and T2DM

It is well established that Kv1.3 channels influence body weight regulation. Kv1.3-KO mice exhibit higher metabolic rates, improved insulin sensitivity, and resistance to diet-induced obesity (Xu et al., 2004). Kv1.3 blockers mitigate the effects of HFD, reducing weight gain and inflammation while improving glucose tolerance in animal models (Upadhyay et al., 2013). In this line, our previous research in the MetS/T2DM model shows that local Kv1.3 blocker treatment after carotid ligation ameliorates vessel remodeling and eliminates HFD-induced insulin resistance and weight gain (Arevalo-Martinez et al., 2021). Interestingly, HFD enhances sensitivity to Kv1.3 inhibition, as Kv1.3 blockade did not affect weight gain in SD mice. Based on these findings, we have explored the contribution of macrophages to the development of MetS, focusing on the potential role of Kv1.3 channels.

Macrophage polarization induces a differential K^+^ channel expression pattern with the upregulation of Kv1.3 channels, which contribute to proliferation, migration and secretion of pro-inflammatory cytokines (Feske et al., 2015; Di Lucente et al., 2018). However, Kv1.3 channel expression and function in macrophages vary significantly depending on factors such as culture conditions, stimulation mode and organ and species source, among others (Nguyen et al., 2017; Simon-Chica et al., 2022).

### 4.3 Contribution of Kv1.3 channels to the MMe phenotype of BMDM

Our data from BMDM from SD and HFD fed mice revealed sex-dependent differences in their susceptibility to develop a metabolically activated (MMe) phenotype. Notably, the MMe marker CD36 was significantly upregulated only in female mice. Similarly, diet-related changes in Kv1.3 channels functional expression were observed exclusively in female BMDM, becoming particularly pronounced upon LPS-induced polarization, with significantly enhanced Kv1.3 upregulation in HFD BMDM (Figure 3). However, the functional impact of this increased Kv1.3 expression in female HFD BMDMs was only apparent when examining their migration rate (Figure 7).

We observed that the combination of diet and LPS induced changes in the phagocytic activity independent of Kv1.3 (Figure 5). The increased phagocytic activity in our MMe BMDMs aligns with previous studies showing CD36 as an important macrophage receptor for apoptotic cell recognition and phagocytosis (Pennathur et al., 2015).

Our metabolic profile analysis indicated that oxygen consumption rate (OCR) remained essentially unchanged across diets (Figure 6). We also found higher extracellular acidification rates (ECAR) in LPS-treated macrophages (Supplementary Figure SIV), indicating an LPS-induced shift towards glycolysis. The glycolytic preference of M1 macrophages has been previously reported in murine models (Zhao et al., 2013; Bess et al., 2023; Gobelli et al., 2023). Interestingly, we also found increased spare respiratory capacity in both SD and HFD LPS-stimulated BMDM (Figure 6), suggesting enhanced oxidative phosphorylation (OXPHOS). While the glycolysis-OXPHOS paradigm has been used to define pro-inflammatory and anti-inflammatory macrophage phenotypes, it is somewhat oversimplified, much like the M1/M2 paradigm. Different pro-inflammatory stimuli mediate different metabolic responses, and different disease states are characterized by different immunometabolic profiles (Mouton et al., 2020). This dual metabolic capability (i.e., increased glycolysis alongside maintained oxidative capacity) has been observed in pro-inflammatory macrophages depending on the activating stimulus (Ishida et al., 2023). Moreover, mouse strain differences can also account for differences in the metabolic response to the same stimulus (Supplementary Figure SV). In parallel experiments using BMDM from BPH and C57 female mice we found an increase in OXPHOS metabolism in response to LPS-treatment in BPH mice, but a decreased OCR in C57 mice.

Regarding HFD treatment, and consistent with our findings, it has been described that in obese mice, adipose tissue macrophages assume a pro-inflammatory phenotype and show both increased glycolytic and OXPHOS metabolism, while peritoneal macrophages do not alter their metabolism, suggesting that the microenvironment drives immunometabolic adaptations during obesity (Boutens et al., 2018). Such metabolic flexibility may allow activated macrophages to adapt to specific environmental conditions, potentially supporting both immediate and sustained inflammatory responses.

### 4.4 Sex-dependent differences in macrophage phenotype in MetS/T2DM mice

Sexual dimorphisms have been documented in immunity; in fact, clinical manifestations of infectious or autoimmune diseases and malignancy differ between females and males, and they are very much dependent on differences in the innate immunity system (Jaillon et al., 2017). A recent study characterized sex differences in the immune system with RNA and ATAC sequence profiling at baseline and after interferon-induced stimulation in 11 immune cell lineages. Surprisingly, only one cell type displayed differences between sexes, namely, the macrophages (Gal-Oz et al., 2019). In agreement with our data (see Table 1), they found that females exhibit a more highly activated innate immune pathways prior to pathogen presentation, and an increased response to interferon stimulus (which can correlate with the response to LPS activation). This female immune alertness makes them less vulnerable to infections but comes at the price of females being more prone to autoimmune diseases. Genes related to lipoprotein metabolism were also upregulated in female macrophages, in agreement with lipid metabolism exhibiting sexual dimorphism (Wang et al., 2011). The stronger inflammatory response in female may contribute to age-related disease developments and life expectancy and can be at the root of the sex-dependent development of the MMe phenotype in our MetS/T2DM model.

### 4.5 Kv1.3 channels as targets against macrophage infiltration in MetS/T2DM

Kv1.3 channels have been shown to contribute to macrophage migration and infiltration in various studies, both *in vivo* and *in vitro*. For instance, Wu et al. (2020) showed that blockage of Kv1.3 with Margatoxin (MgTx) can inhibiting macrophages infiltration in damaged liver tissues. Their *in vitro* studies with RAW264.7 cells suggested that MgTx treatment induced the downregulation of δ-catenin, a protein associated with macrophage migration, indicating that Kv1.3 inhibition represents a potential therapeutic strategy. In the context of CVD, Kv1.3 blockers have been shown to correct AngII induced macrophage infiltration and endothelial dysfunction in small and large vessels (Olivencia et al., 2021). This effect appears to be independent of electrophysiological changes in VSMCs, suggesting a role for Kv1.3 channels in the macrophage-dependent endothelial dysfunction induced by AngII in mice. Kan et al. (2016) proposed a role for Kv1.3 channels in atherosclerosis based on increased Kv1.3 channel expression in macrophages from acute coronary syndrome patients. In RAW264.7 cells, Kv1.3 small interfering RNA suppressed cell migration and reduced ERK1/2 phosphorylation, while Kv1.3 overexpression had the opposite effects. This suggest that Kv1.3 channels may stimulate macrophage migration through activating the ERK1/2-dependent signaling pathway. Interestingly, Kv1.3 induced proliferation in vascular smooth muscle cells and in heterologous expression system also depends on the ERK1/2 signaling pathway (Cidad et al., 2015; 2020; Jiménez-Pérez et al., 2016). Collectively, these findings highlight the importance of Kv1.3 channels in macrophage function and their potential as therapeutic targets in various pathological conditions.

The differences observed between BMDM and PM warrant further analysis. PM, differentiated *in vivo*, typically show modest responses when stimulated *ex vivo*. In contrast, BMDM, differentiated *in vitro*, respond rapidly and robustly to both pro-inflammatory and pro-resolving stimuli, making them the preferred cell type for studying macrophage plasticity (Zajd et al., 2020). BMDM are generally more phagocytic, both in terms of rate and amount of material ingested. They also respond more strongly to polarization, as evidenced by changes in surface molecule expression, gene regulation, and cytokine/chemokine release. For these reasons, we focused our study on the contribution of Kv1.3 to integrated macrophage responses and their changes in MetS/T2DM models using BMDM from female mice.

In our electrophysiological studies comparing both populations, we found that while the ion channel signature was very similar in both cell types, there were differences in the relative amplitude of the components and in their responses to polarization or HFD treatment. However, the low numbers of PM available precluded a thorough characterization of mRNA expression patterns, which would have allowed us to explore expression-function correlations in this cell population. Regarding their role in vascular diseases, it has been reported that PM and BMDM are phenotypically distinct and differ from macrophages in atherosclerotic lesions in terms of M1/M2 marker expression and lipid metabolism genes (Bisgaard et al., 2016). It is important to note that neither PM nor BMDM perfectly match the heterogeneity observed *in vivo*. In fact, their inherent heterogeneity and capacity to polarize rapidly in response to subtle micro-environmental changes may make it impossible to generate a perfect model.

### 4.6 Concluding remarks

Signal-dependent pro-inflammatory stimulation typically activates a broad range of overlapping intracellular cascades. Therefore, the most effective strategies to prevent insulin resistance and T2DM will probably be targeted at proximal and common steps in these pathways. In this scenario, unravelling the mechanisms responsible for monocyte and macrophage migration and infiltration can be relevant for our understanding of the pathophysiological progression to insulin resistance and T2DM. Kv1.3 channels may represent a promising therapeutic target for mitigating inflammation-driven metabolic disorders. By interfering with macrophage migration, Kv1.3 inhibition could potentially disrupt the vicious cycle of inflammation and insulin resistance, offering a novel approach to managing MetS and preventing its progression to T2DM and its associated CV complications. In addition, our findings reveal important sex-specific differences in macrophage function, which can contribute to design more effective and personalized interventions.

## Data Availability

The raw data supporting the conclusions of this article will be made available by the authors, without undue reservation.
