# Supplementary material for: A sex-dependent role of Kv1.3 channels from macrophages in metabolic syndrome
Source: Front Physiol. 2024 Nov 13;15:1487775. doi: 10.3389/fphys.2024.1487775 (PMC11599228; doi:10.3389/fphys.2024.1487775)
Supplement: Supplementary file 1 [file Supplementaryfile1.docx]

# Supplemental Material

## Supplemental Table 1.

| Table I: Primers used in RT-QPCR experiments | | | |
| --- | --- | --- | --- |
| **Protein** | ***Gene*** | ***Assay*** | ***Sequence*** |
| **K_V_1.3** | Kcna3 | Taqman^®^ | **F:** 5'-CCACGAACAATAACCCCAAC-3'  **R:** 5'-AGGGCATACACAGACCAAGG-3'  **Probe**: 5'-GTCTAATATATGATACGGTTGCCA-3' |
| **K_Ca_3.1** | Kcnn4 | Taqman^®^ | Mm00464586_m1 |
| **K_ir_2.1** | Kcnj2 | Taqman^®^ | Mm00434616_m1 |
| **P2X_7_** | P2rx7 | Taqman^®^ | Mm01199500_m1 |
| **TNF-α** | Tnf | Sybr Green | **F:** 5'-AGCCGATGGGTTGTACCTTGTCTA-3'  **R:** 5'-TGAGATAGCAAATCGGCTGACGGT-3' |
| **NOS2** | Nos2 | Sybr Green | **F:** 5'-CCAAGCCCTCACCTACTTCC-3'  **R**: 5'-CTCTGAGGGCTGACACAAGG-3' |
| **CD36** | Cd36 | Sybr Green | **F:** 5'- ATGGGCTGTGATCGGAACTG-3'  **R:** 5'-GTCTTCTCAATAAGCATGTCTCC-3' |
| **RPL18** | Rpl18 | Taqman^®^ | **F:** 5'-AAGACTGCCGTGGTTGTGG-3'  **R:** 5'-AGCCTTGAGGATGCGACTC-3'  **Probe:** 5'-FAM-TTCCCAAGCTGAAGGTGTGTGCA-BHQ1-3' |

Primers and probes used in RT-QPCR experiments.

## Supplemental Figure I

**Graphical representation of data from Table 1.** mRNA expression levels of several phenotype markers and ion channels were determined with qPCR in BMDM from male (gray scale bars) and female mice (red scale bars) subjected to SD or HFD as labelled. mRNA levels are expressed as normalized abundance (2^-ΔCt^) using Rpl18 as the housekeeping gene. Statistical comparisons were carried out using two-way ANOVA followed by Tukey’s test in the case of normal distributions and equal variances; alternatively Kruskal-Wallis analysis followed by Dunn’s test was used. P values in blue are significant differences between the corresponding bars in males and females. Values are mean±SEM of 8-12 triplicate determinations, obtained from two batches of SD and HFD mice in each group (male and female).

## Supplemental Figure II

**Flow cytometer analysis of CD36 expression in BMDMs. A**. The presence of CD36 in the membrane was evaluated in female BMDM obtained from SD and HFD-fed mice both under resting conditions (control) and after LPS treatment for 16 h (LPS) using flow cytometry. BMDMs in suspension were stained with Super Bright 780-conjugated anti-CD36 antibody (eBioscience^TM^) at 1:100 and the cells were analysed using an Aurora flow cytometer (Cytek Biosciences). The bars represent de media+esm of the median fluorescence intensity of 3-4 independent experiments from different mice, each one containing 3 replicates. Statistical significance was obtained with a two-way ANOVA followed by Tukey’s post-hoc test. **B.** Representative histograms of the median fluorescence obtained for resting BMDMs on SD and HFD treatment.

## Supplemental Figure III

**Electrophysiological characterization of outward K^+^ currents from PM.** Kinetic and pharmacological studies were carried out in PM obtained from male ( grey scale colors, upper plots) and female mice (lower plots, red scale colors). In all cases, BMDM obtained from SD and HFD-fed mice were studied at rest (M0) and after16 h treatment with 100 ng/ml LPS (M1) as indicated. **A. B.** Current density to voltage relationships were constructed from peak outward current amplitudes obtained in 200 ms pulses from -80 to +60 mV in 10 mV steps, from a holding potential of -80 mV. Data are mean±SEM of 25-40 cells in each group, obtained from at least 8 different animals. **C.D.** Peak current amplitude was obtained from trains of 40 250 ms pulses from -80 mV to +40 mV applied every 0.5 s, and normalized to the amplitude of the first pulse. The symbol graphs show the mean±SEM of the first 20 pulses fitted to a one-exponential decay function, and the bars plots on the right were obtained by averaging the normalized amplitude (expressed as %) of the last 20 pulses in each group. P values were obtained from F-test comparison between the fits (for the normalized current plot) and with one-way ANOVA for the % of UDB. Data are mean±SEM of 25-35 cells per group, from at least 8 different cultures. **E.F.** Average peak current amplitude for the PAP-1 sensitive (Kv1.3 current), the TRAM34-sensitive (K_Ca_3.1 current) the 5 mM TEA-sensitive (Kv2-Kv3 current) and the insensitive outward K current (unblock) in each of the conditions 13-25 PM obtained from at least 8 different animals were used for each determination.

## Supplemental Figure IV.

**Seahorse Cell Mito Stress Test**. Average acidification rate data (ECAR) were measured in real-time using a Seahorse XF24 Analyzer (Agilent, California, CA, USA) with Seahorse XF Cell Mito Stress Test experiments carried out in BMDM from SD and HFD female mice, using resting (M0) and activated (M1) cells with or without overnight treatment with 100nM PAP-1 as indicated. Data were obtained from 3 independent experiments, each one containing 3 replicates.

## Supplemental Figure V

**Seahorse Cell Mito Stress Test C57BL/6J vs. BPH**. Average data from Seahorse XF Cell Mito Stress Test experiments carried out in BMDM from C57BL/6J and BPH female mice, using control and LPS-activated macrophages. The Cell Mito Stress Test profile was obtained by sequential application of the drugs (see methods for details). Data was normalized by counting of cells and expressed as pmol O_2_/min/1000 cells. Data were obtained from 3 independent experiments, each one containing 3 replicated and analyzed with a 3-way ANOVA followed by Tukey’s post-hoc test.
